# The Path Toward PET-Guided Radiation Therapy for Glioblastoma in Laboratory Animals: A Mini Review

**DOI:** 10.3389/fmed.2019.00005

**Published:** 2019-01-29

**Authors:** Sam Donche, Jeroen Verhoeven, Benedicte Descamps, Julie Bolcaen, Karel Deblaere, Tom Boterberg, Caroline Van den Broecke, Christian Vanhove, Ingeborg Goethals

**Affiliations:** ^1^Department of Radiology and Nuclear Medicine, Ghent University, Ghent, Belgium; ^2^Department of Pharmaceutical Analysis, Ghent University, Ghent, Belgium; ^3^Department of Electronics and Information Systems, Ghent University, Ghent, Belgium; ^4^Department of Radiation Oncology and Experimental Cancer Research, Ghent University, Ghent, Belgium; ^5^Department of Pathology, Ghent University, Ghent, Belgium

**Keywords:** PET, radiation therapy, laboratory animals, dose painting, glioblastoma, tumor heterogeneity

## Abstract

Glioblastoma is the most aggressive and malignant primary brain tumor in adults. Despite the current state-of-the-art treatment, which consists of maximal surgical resection followed by radiation therapy, concomitant, and adjuvant chemotherapy, progression remains rapid due to aggressive tumor characteristics. Several new therapeutic targets have been investigated using chemotherapeutics and targeted molecular drugs, however, the intrinsic resistance to induced cell death of brain cells impede the effectiveness of systemic therapies. Also, the unique immune environment of the central nervous system imposes challenges for immune-based therapeutics. Therefore, it is important to consider other approaches to treat these tumors. There is a well-known dose-response relationship for glioblastoma with increased survival with increasing doses, but this effect seems to cap around 60 Gy, due to increased toxicity to the normal brain. Currently, radiation treatment planning of glioblastoma patients relies on CT and MRI that does not visualize the heterogeneous nature of the tumor, and consequently, a homogenous dose is delivered to the entire tumor. Metabolic imaging, such as positron-emission tomography, allows to visualize the heterogeneous tumor environment. Using these metabolic imaging techniques, an approach called *dose painting* can be used to deliver a higher dose to the tumor regions with high malignancy and/or radiation resistance. Preclinical studies are required for evaluating the benefits of novel radiation treatment strategies, such as PET-based *dose painting*. The aim of this review is to give a brief overview of promising PET tracers that can be evaluated in laboratory animals to bridge the gap between PET-based *dose painting* in glioblastoma patients.

## Introduction

Brain tumors are relatively rare when compared with breast, lung, prostate, and colorectal cancer, however, malignant brain tumors are among the most feared types of cancer. Besides poor prognosis, these tumors have a direct impact on quality of life and cognitive function (1). Tumors originating from glial cells, the so-called gliomas (2), can be classified into low-grade gliomas (LGG, WHO grade I-II) and high-grade gliomas (HGG, WHO III-IV). Glioblastoma (GB, WHO IV) is the most aggressive and malignant primary brain tumor. Usually, GB is a solid tumor that can be characterized by infiltrative boundaries, heterogeneous composition, and hemorrhage (3). In contrast to WHO I to III gliomas, GB exhibits microvascular proliferation and necrosis as a defining feature (4). GB is also characterized by disruption of the blood-brain barrier, which is responsible for leakage of gadolinium-based agents in contrast-enhanced magnetic resonance imaging (MRI) (5). The infiltrative growth often delays early diagnosis until symptoms from mass effect arise. It also renders a complete surgical resection nearly impossible without causing significant neurological injury. Hence, residual glioma cells at the tumor margins frequently lead to tumor recurrence (6).

Despite the discovery of several novel therapeutic targets for chemo- and immunotherapy (7), none have proven to be effective due to the anatomically and immunologically nature of the brain (8, 9). Consequently, the treatment for GB patients has stagnated since the introduction of the Stupp protocol in 2005, which consists of maximal surgical resection followed by combined external beam radiation therapy (RT) and concomitant temozolomide, followed by adjuvant temozolomide for newly diagnosed GB patients with a good performance status (10). Therefore, alternative treatment approaches are necessary. There is a well-known dose-response relationship for glioblastoma with increased survival with increasing doses, but this effect seems to cap around 60 Gy, due to increased toxicity to the normal brain (11). As a result, additional information from metabolic imaging techniques, such as positron-emission tomography (PET), for target volume definition during radiotherapy planning is a reasonable option. These techniques enable the visualization of biological tumor features *in vivo* and may facilitate customization of dose prescription.

Since only a limited amount of information can be obtained through clinical trials and because it has been hypothesized that a better understanding can be obtained from downscaling to small animals (12, 13), preclinical studies using precision image-guided radiation research platforms (14) are relevant for investigating current unresolved challenges in radiation oncology toward personalized medicine and novel treatment strategies, such as dose painting (15). In addition, certain experimental setups (e.g., autoradiography) cannot be performed in the clinic. The purpose of most preclinical RT studies is to translate discovery to human trials and preclinical RT studies should be designed to flow over into a Phase I clinical trial (15). On the other hand, preclinical research can be carried out in parallel with or subsequent to clinical trials to gain *de novo* understanding about trial conclusions (16). However, preclinical data must be interpreted accurately and limitation of these preclinical setups have to be considered (15). The aim of this review is to give a brief overview of promising PET tracers that can be evaluated in laboratory animals to bridge the gap between PET-based dose painting in glioblastoma patients.

## Radiation Therapy Planning

### Biological Target Volume

Neuroimaging is of major importance for RT planning. Shortly after its introduction, computed tomography (CT)-based conformal RT planning was incorporated into the standard of care for cancer patients. Whereas, MRI provides superior tumor visualization, CT remains fundamental for dosimetry, and imaging dose-limiting organs (17). On CT and conventional MRI, two main tumor volumes for RT planning are delineated: the gross tumor volume (GTV), identifying the position and extent of the macroscopic gross tumor, and the clinical target volume (CTV) that contains the GTV plus a margin for sub-clinical disease spread, which cannot be fully imaged, and is crucial for maximization of the radiation dose to the tumor. Subsequently, another margin is incorporated to account for setup and delivery uncertainties to obtain the planning target volume (PTV) (18, 19). Currently, GTV for GB is determined by T_1_-weighted contrast-enhanced MRI and T_2_/fluid-attenuated inversion recovery (FLAIR) sequences. An isotropic expansion of these margins results in the CTV (20).

In 2000, an additional concept was introduced, namely the biological target volume (BTV) that can be derived from functional or molecular imaging techniques, such as PET. For example, the tumor burden or hypoxic tumor region obtained through magnetic resonance spectroscopy (MRS) and [^18^F]FMISO (see further), respectively, are important to consider when planning RT (21). It was suggested by Navarria et al. that BTV may lead to a more accurate delineation of the CTV as tumor recurrences are often situated in this region (22). It was also shown that the volume of BTV is correlated with the overall survival in GB patients (23). With the increased availability of metabolic information and appreciation for tumor heterogeneity (see further), radiation oncologists started to consider an evolution from the traditional concept of a uniform dose distribution toward a non-uniform dose distribution (24).

### Dose Painting

**Figure 1:**
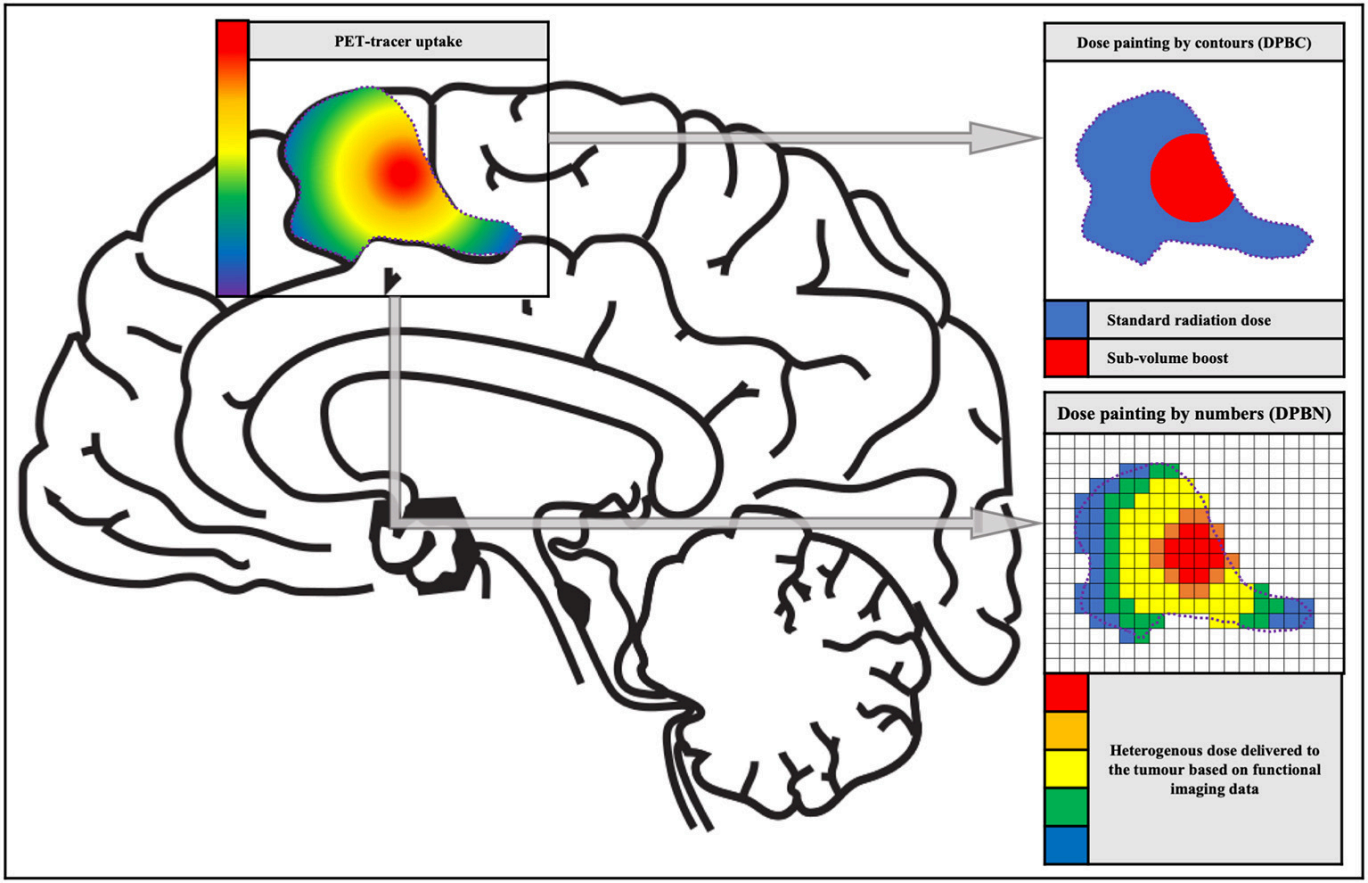
The concept of dose painting. Schematic representation of the two dose painting methods: dose painting by contours (DPBC) and dose painting by numbers (DPBN). The image and color bar on the left show the PET tracer uptake. The images and color scales on the right display a discrete fictive dose distribution for radiation therapy.

In the majority of HGG, intratumor heterogeneity is established through the diverging genetic drift of tumor subclones. These subclones respond to the therapy to a varying degree and are often spatially segregated (25). Fast dividing tumor clones can be associated with proliferation corresponding with highly malignant tumor regions. Hypoxic tumor regions can be associated with reduced oxygenation. This oxygen deficiency is a *primum movens* for the development of radiation resistance or radiation insensitivity, which in turn is the basis for tumor recurrence (25). This additional information on biological tumor variation can be integrated into radiotherapy planning in order to facilitate heterogeneous radiation therapy (26). In 2000, Ling et al. introduced the term “dose painting” in a review paper on multidimensional radiotherapy (21). The concept of dose painting is to “paint” an increased radiation dose on tumor volumes with more radiation resistance and/or malignancy. Dose painting can be accomplished in two ways: dose painting by contours (DPBC) and dose painting by numbers (DPBN), whereby a dose is given to a set of nested sub-volumes or at voxel level, respectively (25) (see Figure 1).

When DPBC (also called sub-volume boosting) is utilized, a sub-volume within the GTV is treated with a higher (uniform) dose compared with the rest of the PTV to obtain an improved treatment outcome (27, 28). A drawback of discrete volumes is that they are binary, which means voxels are either inside or outside the volume. However, in biological reality, one can observe gradients in hypoxic tissue, cellular phenotypes and malignancy, and by differentiating only one sub-volume a substantial amount of information is lost. This discrepancy has led to the development of dose painting by numbers (25).

In DPBN, the dose for each voxel is calculated incorporating the intensity of the corresponding voxel in e.g., a PET image. This method often uses a lower and upper boundary, to make sure that enough dose is delivered to every part of the tumor while protecting the organs at risk. The most basic method is a linear interpolation between the minimum and maximum dose, proportional to the minimum and maximum intensity within the target volume (29). However, there is some skepticism about the quality assurance of DPBN, which might have an increased risk of radiation-induced secondary cancers (25).

The most optimal dose painting approach still has to be demonstrated through (pre)clinical research. For instance, a prospective phase II study evaluated the integrated boost intensity-modulated dose escalation concept using [^18^F]FET to obtain a better local tumor control. However, the results showed that dose escalation did not lead to a survival benefit (30).

## PET Imaging in Glioblastoma

As mentioned above, RT planning is critically dependent on neuroimaging. MRI and CT represent the two most important and commonly used imaging modalities. The former is the method of choice for assessment of tumor volume and location while the latter is mandatory for RT planning. However, despite the remarkable soft tissue contrast of (conventional) MRI, it offers a limited grasp on malignancy grade, infiltration into the surrounding normal tissue, tumor heterogeneity, and differentiation between (radio)necrosis and recurrent tumor (20). For instance, tumor cells can be found at a cm range from the contrast enhancing tumor part on MRI (31, 32). Furthermore, pseudo-progression and pseudo-response (image alterations due to therapy rather than tumor evolution) complicate response assessment in glioma using conventional MRI (33, 34).

Molecular imaging techniques, such as PET, provide additional information on tumor biology. PET may have an impact on tumor delineation for RT planning because increased tracer uptake after surgery can often be found outside the contrast-enhancing region or the T_2_/FLAIR tumor volume on MRI (35). Thus, by incorporating PET imaging into RT planning an improved local tumor control and a reduced exposure of healthy tissue can be obtained (5). In addition, biological changes may precede anatomical changes after the start of therapy. This information can serve different purposes, such as more accurate diagnosis, biopsy guidance, and adaptive radiation treatment (36, 37). In the past decades, a variety of tracers have been developed as imaging agents for different metabolic pathways of neuro-oncologic cells that might be promising for PET-based radiation treatment.

### [^18^F]Fluorodeoxyglucose PET

The most (pre)clinically used PET tracer in oncology is 2-deoxy-2-[^18^F]fluoro-d-glucose([^18^F]FDG) because it has a high potential to detect tumors in the body based on the increased energy (glucose) requirements of malignant tumors (5, 38). However, localization and delineation of (primary) brain tumors is often difficult due to the high background glucose metabolism of normal brain parenchyma. Only coregistration of [^18^F]FDG PET with MRI allows accurate assessment of glucose metabolism in specific areas of the tumor (5, 36, 39). Despite this phenomenon, it is worth mentioning that it has been demonstrated that delayed [^18^F]FDG imaging (3–8 h after tracer injection) improves the distinction between tumor and normal gray matter because the washout of glucose is higher in normal brain tissue than in tumor tissue (39, 40). Although [^18^F]FDG PET-guided radiation therapy is routinely used to treat other cancer types, e.g., head-and-neck cancer (41), PET guidance using amino acid tracer seems to be more suitable for these procedures (42, 43).

### Amino-Acid PET

Due to their relatively low uptake in normal brain parenchyma (5) and low variability in delineation amongst operators (44), radiolabelled amino acids, and amino acid analogs are the most commonly used PET tracers for neuro-oncological imaging (39, 45).

In the early 1980s, l-(methyl-[^11^C])-methionine ([^11^C]MET) was introduced as PET tracer for imaging brain tumors (46, 47). Over several decades, [^11^C]MET PET has demonstrated its value in the initial diagnosis and image-guided biopsy (42, 48, 49), the detection of tumor recurrence (50, 51), tumor prognosis (52), and RT planning (5, 53–55).

Although most PET studies of gliomas are performed with [^11^C]MET, the short half-life of the radioisotope [^11^C] (~20 min) is a major drawback, which necessitates the presence of a cyclotron nearby the clinical facility. Therefore, amino acid (analog) PET tracers labeled with radioisotopes with a longer half-life were developed, e.g., O-(2-[^18^F]fluoroethyl)-l-tyrosine ([^18^F]FET) and 3,4-dihydroxy-6-[^18^F]fluoro-l-phenylalanine ([^18^F]FDOPA) labeled with [^18^F] (half-life ~110 min). Previous studies have shown that both [^18^F]FET and [^18^F]FDOPA provide analogous (diagnostic) information compared to [^11^C]MET PET in glioma patients (44, 56).

Currently, [^18^F]FET is the preferred clinical tracer for brain tumors and its diagnostic potential is well-documented (36, 57, 58). The vast majority of HGG show increased [^18^F]FET uptake. However, the absence of [^18^F]FET uptake does not exclude the diagnosis of glioma, since a considerable number of LGG are [^18^F]FET negative (59). The superior delineation of [^18^F]FET PET for glioma patients in biopsy and RT guidance in comparison with MRI was repeatedly shown (45, 60, 61). [^18^F]FET PET has also been used for the definition of an integrated boost to residual tumor after initial surgery (30) and recurrent tumor (62).

**Figure 2:**
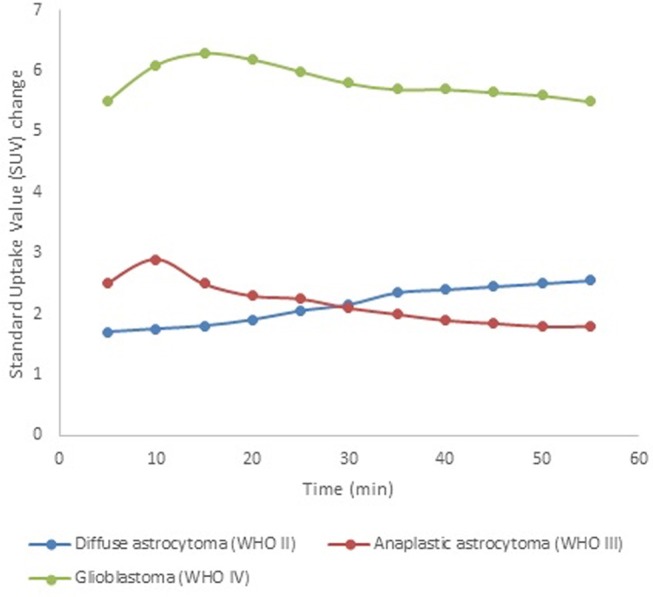
[^18^F]FET time-activity curves for tumor grade assessment. These simulated data show typical examples for diffuse astrocytoma (WHO II, blue), anaplastic astrocytoma (WHO III, red), and glioblastoma (WHO IV, green) on dynamic [^18^F]FET PET scans. This illustrates the discrepancy between LGG, which typically show a steadily increasing time-activity curve, and HGG (WHO III-IV), which typically show an early peak followed by a washout period.

Furthermore, in addition to static images, dynamic [^18^F]FET PET data can be acquired, providing significantly more information on both temporal and spatial tracer uptake. The time-to-peak and the shape of the [^18^F]FET time-activity curve have been shown valuable for patient care (63). For example, tumor grading accuracy can be substantially improved through the assessment of dynamic [^18^F]FET PET data, which typically show steadily increasing time-activity curves in WHO grade II gliomas, as opposed to an early activity peak (~10–20 min after injection), followed by a decrease of [^18^F]FET uptake in WHO grade III/IV gliomas (see Figure 2) (64, 65). These data have proven to be important for therapy response assessment (66), in differentiating progressive or recurrent glioma from treatment-related non-neoplastic changes (67), and in the assessment of prognosis (68–70).

### Hypoxia PET

Tumor oxygenation has an essential role when considering resistance to radiation therapy. In 1955, the negative effect of tumor hypoxia on tumor outcome was demonstrated (71). Inadequate oxygen supply results in changes in metabolism and cellular proliferation (72), which results for example in a required radiation dose up to three times higher than the dose for well-oxygenated tissues (5, 73). Detection of this phenomenon in tumors has a high clinical relevance because tumor aggressiveness, metastatic spread, failure to achieve local tumor control, increased rate of recurrence, and ultimately poor outcome are all associated to hypoxia (5, 72, 74, 75).

The first developed hypoxia PET tracer was [^18^F]fluoromisonidazole ([^18^F]FMISO). [^18^F]FMISO can passively diffuse through the membrane and binds covalently to intracellular proteins under hypoxic conditions, resulting in tracer accumulation within hypoxic cells (5, 36, 38, 73, 76). With regard to therapy response assessment, the volume and the intensity of hypoxia signal on [^18^F]FMISO PET in GB before radiation therapy was strongly correlated with poor progression and survival (39, 77).

The slow uptake of [^18^F]FMISO in target tissue and slow clearance of unbound [^18^F]FMISO from non-hypoxic areas has led to the development of [^18^F]fluoroazomycin-arabinoside ([^18^F]FAZA) with improved pharmacokinetics (74, 78). In the majority of gliomas, a clear distinction between hypoxic tumor tissue and normal parenchyma could be observed 2 h after intravenous injection of [^18^F]FAZA (79). Nevertheless, further research is needed before the abovementioned hypoxia tracers can be incorporated in PET-guided RT for glioma patients.

## Small Animal PET-Guided Radiation Therapy

For many decades, laboratory animal radiation research was mostly performed using fairly crude experimental setups (14). The delivery of radiation in small animals was achieved using fixed radiation sources (80–82) or linear accelerators producing megavoltage X-rays (83–85) and applying only a single radiation field (82–84, 86). This approach often results in full/partial body irradiation or in the best case at a precision of a few mm, while sub-millimeter precision is required for small animals (14). Furthermore, simple single-beam techniques were commonly used without the ability to target a specific tumor volume, hampering response assessment due to high doses delivered to healthy brain tissue (14, 82, 84, 85, 87). These techniques significantly differ from the advanced 3D image-guided radiotherapy techniques using conformal arcs in clinical practice (13).

To enable more accurate (conformal) irradiation in laboratory animal research, precision image-guided small animal radiation research platforms were developed. These platforms typically integrate a kV X-ray source that is used for imaging and radiation treatment, a computer-controlled stage for animal positioning, a rotational gantry assembly to allow radiation delivery from various angles, and a collimating system to shape the radiation beam. The Small Animal Radiation Research Platform (SARRP, XStrahl^®^, Surrey, UK) (13), developed at Johns Hopkins University School of Medicine, uses a 225 kV X-ray tube that is mounted on a motorized arm that rotates around an animal stage that can displace in three orthogonal directions and rotate around the vertical axis. A “fixed” on-board flat panel detector allows for cone beam computer tomography by rotating the animal stage around its vertical axis. Nozzle-shaped or a motorized variable collimator provides circular and rectangular radiation fields with different dimensions. The X-RAD 225Cx (Precision X-Ray Inc., North Branford, US) (88) from Princess Margeret Hospital uses the same X-ray tube as the SARRP, however, the animal stage is fixed, and X-ray tube and detector panel rotate around the stage. Different circular and rectangular beam sizes can be used during irradiation. At the University of Texas Southwestern, a fixed high energy X-ray tube of 320 kV is combined with a fixed imaging panel. The principle of the animal stage is similar to the one of the SARRP, except the fact that it rotates around its horizontal axis instead of its vertical axis (89, 90). A group at Stanford University modified the eXplore RS120 microCT scanner to use it as a small animal radiation platform with an excellent spatial imaging resolution. Pseudo-circular radiation fields are produced by an iris-shaped collimator to deliver beams at 120 kV. The system has its own treatment planning system and produces small penumbras for small fields, nevertheless heating problems, and a low efficiency to deliver high doses prevented the system from being commercialized (91). Finally, the image-guided Small Animal Arc Radiation Treatment system (iSMAART) from the University of Miami (92–94) consists of an X-ray source, a flat panel detector, a charge-coupled detector (CCD), and an animal stage capable of rotating and x-y-z translation. All the components remain stationary except the rotating animal stage, which allows to acquire CT, tomographic bioluminescent, and tomographic fluorescent images for guided treatment delivery. The development and commercialization of small animal image-guided radiotherapy devices has decreased the technological gap with clinical RT. The number of preclinical trials using these devices for precision small animal RT has been steadily increasing over the past years. Now, researchers are capable to conduct preclinical investigations in a manner that more closely resembles the clinical scenario and these devices have the potential to address current challenges regarding PET-based dose painting strategies (15, 95).

However, while dose calculations in the clinic are done by inverse treatment planning, whereby one starts from a desired dose distribution to calculate the beams via an (iterative) algorithm, small animal irradiators often function with forward planning. In forward planning, the radiotherapy planner selects the number and angle of beams. The computer then calculates the dose distribution. The plan is optimized by manual iteration, which is labor intensive (96). From 2009 and onwards, research efforts have been made to implement inverse planning on these research platforms as well (97–99). To further increase conformity with the clinical situation, a motorized variable rectangular collimator was developed as a preclinical counterpart of the multi-leaf collimator. In 2014, Cho et al. presented a 2D dose painting method using this variable collimator (100).

Recently, the same group implemented a 3D inverse treatment planning procedure on a micro-irradiator and defined a minimum dose for the target volume and a maximum dose for the OAR (101). These techniques have mostly been evaluated *in silica* and application in laboratory animals still needs to be investigated.

Similar to the clinical situation, treatment planning on these radiation research platforms is based on CT (102). However, (preclinical) CT is hampered by insufficient soft-tissue contrast, which makes brain tumor localization very challenging. To improve target selection, CT on these preclinical research platforms is increasingly being combined with functional imaging modalities, such as PET and bioluminescent imaging (38).

The implementation of PET for RT planning is still under investigation in the clinic (103–105) and preclinical research might provide new insights for combining PET with RT. Evidently, PET-based RT planning requires correct registration with the planning CT to obtain accurate treatment planning. This process can be simplified by using a multi-modality bed to move the animal from the PET to the micro-irradiator and, ideally, a (semi-) automatic registration algorithm should be used to minimize intra- and inter-observer variability. The major weaknesses of PET imaging for RT planning are the relatively long acquisition times, the high cost of a PET scanner and limited spatial resolution (1–2 mm range). Moreover, integrating a PET device into a preclinical radiation research platform is far from trivial. These limitations might be related to the success of optical molecular imaging techniques to guide RT, such as bioluminescence and fluorescence imaging. Optical imaging is free of ionizing radiation, is a relatively inexpensive imaging technique with short acquisition times and the compact footprint enables it to be integrated into a micro-irradiator. Several groups have demonstrated the feasibility to integrate optical imaging into a micro-irradiator (93, 94, 106–108). Related to image guidance, bioluminescent imaging provides excellent signal-to-background ratios due to the negligible background signal, while various fluorescent probes are available for tumor-specific target imaging. However, optical imaging suffers from absorption and scattering of visible light by tissue, limiting the spatial resolution and the accuracy to localize a target. Fluorescence imaging is also hampered by auto-fluorescence, resulting in a background artifact, and it should be noted that bioluminescent imaging is difficult to translate to the clinic because it requires genetic manipulations of tumor cells for *in vivo* applications.

Related to PET-guided RT, only a few promising studies have been carried out. In 2011, [^18^F]FET PET guidance has been used in boron neutron capture therapy, an alternative radiation treatment approach, in F98-tumor bearing rats (109). In 2015, the use of BTV in a preclinical setup has been positively evaluated by Trani et al. in rat rhabdomyosarcomas (110). Recently, our neuro-oncology research group incorporated PET-based sub-volume boosting in the preclinical workflow for RT planning for both [^18^F]FET and [^18^F]FAZA (111) and this methodology was applied to investigate treatment outcome in a rat model of GB [unpublished data].

## Conclusion

Despite research efforts, the treatment of GB patients has stagnated since the introduction of the Stupp protocol. Therefore, novel therapeutic approaches should be investigated for this cancer with a poor prognosis. Routine diagnosis and treatment planning of GB patients is still heavily dependent on contrast-enhanced MRI. Literature has shown the assets of various PET tracers in the different steps of patient care: detection, grading, differentiating tumor recurrence from radionecrosis, prognosis, and therapy response assessment. PET also has the potential to improve tumor delineation for RT due to its capabilities to visualize radiation resistance and/or malignant tumor tissue. The feasibility of PET-based radiation therapy has been clinically investigated for different tracers. It is our opinion that PET should be considered for RT planning of GB because of valuable biological information. Preclinical models have a supporting function toward developing clinical applications, e.g., examination of novel dose painting strategies and radiobiological hypotheses or correlations to histopathology. Also, further development of the preclinical models is still necessary to reach the same degree of complexity and accuracy as their clinical counterpart.

## Author Contributions

IG and CV designed and developed the concept of the manuscript. SD wrote the manuscript. IG, CV, JV, BD, JB, TB, CVdB, and KD supervised and edited the manuscript. SD prepared Figures 1, 2. All authors discussed and commented on the manuscript at all stages.

### Conflict of Interest Statement

The authors declare that the research was conducted in the absence of any commercial or financial relationships that could be construed as a potential conflict of interest.
